# Topological bio-scaling analysis as a universal measure of protein folding

**DOI:** 10.1098/rsos.220160

**Published:** 2022-07-13

**Authors:** Sergey Shityakov, Ekaterina V. Skorb, Michael Nosonovsky

**Affiliations:** Infochemistry Scientific Center (ISC), ITMO University, 9 Lomonosova St., St Petersburg 191002, Russia

**Keywords:** Trp-cage, folding, ergodicity, scaling, fractal dimension

## Abstract

Scaling relationships for polymeric molecules establish power law dependencies between the number of molecular segments and linear dimensions, such as the radius of gyration. They also establish spatial topological properties of the chains, such as their dimensionality. In the spatial domain, power exponents *α* = 1 (linear stretched molecule), *α* = 0.5 (the ideal chain) and *α* = 0.333 (compact globule) are significant. During folding, the molecule undergoes the transition from the one-dimensional linear to the three-dimensional globular state within a very short time. However, intermediate states with fractional dimensions can be stabilized by modifying the solubility (e.g. by changing the solution temperature). Topological properties, such as dimension, correlate with the interaction energy, and thus by tuning the solubility one can control molecular interaction. We investigate these correlations using the example of a well-studied short model of Trp-cage protein. The radius of gyration is used to estimate the fractal dimension of the chain at different stages of folding. It is expected that the same principle is applicable to much larger molecules and that topological (dimensional) characteristics can provide insights into molecular folding and interactions.

## 1. Introduction

The function of most proteins, with the exception of intrinsically disordered proteins and intrinsically disordered regions (IDRs), is defined by their unique structure encoded in amino acid (AA) sequence. The folding of proteins defines their structure, and it is pivotal for their functional properties. However, the simulation of protein folding remains a difficult problem. Numerous algorithms and methods of the simulation of folding have been suggested [1–4]; however, they all have comparative advantages and disadvantages in terms of their computational time cost, applicability to dynamics of transitional states (TS) and diverse molecular structures, and precision (table 1).

**Table 1.** Comparative characteristics of methods of protein folding simulation.

method	time	dynamics (TS)	structure	precision (RMSD, Å)
topological bioscaling analysis	super-fast	applicable	various biomolecules (proteins, polymers, etc.)	depends on external data
ML ‘AlphaFold’	fast	not applicable	only protein molecules	0.6–1.5 [1]
Monte Carlo ‘Rosetta’	relatively fast	not applicable	only protein molecules	2.6 [2]
MD [3]	slow	applicable	various biomolecules (proteins, polymers, etc.)	2.5–3.0
DFT [4]	extremely slow (not applicable)	applicable	small organic molecules (drugs, ligands, etc.)	not applicable for folding

Thus, among the traditional algorithms, the density functional theory (DFT) methods, while precise, are only applicable to small molecules due to their high time consumption. Since the computation of wave function is very time-consuming, the DFT cannot be used currently for protein folding simulation in most cases.

The molecular dynamics (MD) simulation is a general method that can be used for the study of the folding of various biomolecules. On the other hand, MD is applicable for protein folding and enables visualization of the TS structures dynamically. On the other hand, the method is relatively slow and time-consuming for a large number of rotational degrees of freedom.

The Monte Carlo (Rosetta) simulation is a faster method of folding analysis, which is applicable to proteins. However, it requires a reference structure (usually obtained crystallographically) to calculate protein folding.

Novel machine learning (ML)-based methods, such as the ‘AlphaFold,’ are becoming increasingly popular for the folding simulation. They are relatively fast and precise, although not applicable to the TS analysis.

However, qualitative information about folding can also be obtained from the mathematical methods of topological analysis of molecular chains. At various stages of folding, molecular chains can be viewed as complex linear (one-dimensional) or three-dimensional structures. Here, we will suggest a simple method based on topological analysis of scaling properties, which can reveal such characteristics as the effective dimensionality of a molecule. The method is not time-consuming, and it provides very fast insight into the folding stage, applicable to both biomolecules and polymers.

## 2. Scaling properties of polymer molecules

Many properties of long polymer molecules can be related to their scaling behaviour. Scaling relationships establish dependencies between the number of segments in the molecule, *N*, the length of the individual segment, *a*, and the end-to-end distance

2.1 $$L∼aN^{\alpha },$$

where *α* is the so-called scaling exponent.

The simplest ideal chain model of a polymer molecule implies the uniformly distributed random orientation of neighbouring segments. The average end-to-end distance, 〈*L*〉, scales as the random walk, so that the average distance is proportional to the square root of the number of segments

2.2 $$\langle L\rangle =aN^{1/2}.$$

More realistic polymer chain models take into account (i) the energy cost associated with segment rotation instead of assuming the uniformly distributed random orientation of neighbouring segments and (ii) the steric effect of the excluded volume. To account for the non-uniform distribution of angles between neighbouring segments, the so-called effective Kuhn length of a segment is introduced, which still provides the scaling relation of equation (2.2) with *α* = 1/2 [5].

To account for the excluded volume, a model of the non-intersecting chain is used. According to the Flory theory, a certain excluded volume *v* is associated with every segment. The total of *N* segments occupy the volume *L*^3^. The energy of the molecule involves two components: the repulsive energy between segments and the elastic energy of the chain. The repulsive energy per segment is proportional to the volume ratio *Nv*/*L*^3^ and thus the total repulsive energy involves *N*^2^*v*/*L*^3^. The elastic energy of interaction with the solvent is proportional to (*L*/〈*L*〉)^2^, so that the total free energy is given by

2.3 $$F(L)∼\frac{N^{2}v}{L^{3}}+\frac{L^{2}}{Na^{2}}.$$

The minimization of *F*(*L*) yields

2.4 $$\frac{dF}{dL}\;∼\frac{2L}{Na^{2}}-3\frac{N^{2}v}{L^{4}}=0,$$

which immediately provides a scaling relation between the number of segments and the end-to-end distance

2.5 $$L=N^{3/5}{\left(\frac{3v}{2a^{2}}\right)}^{1/5}.$$

While equation (2.5) yields *α* = 0.6, more accurate estimates using the renormalization group theory result in the value of *α* = 0.588 [6].

A common way to modify the scaling behaviour of molecules is to adjust their solubility, for example, by tuning temperature. With increasing temperature, solubility tends to grow, and non-soluble globular molecules would dissolve and unfold. According to the Flory theory, during the coil-globule transition, the scaling exponent would pass the entire range between the value of a compact globule to that of the coil. The transition point when the scaling exponent is *α* = 1/2 is called the *theta-point* or *theta-transition*. At the theta-point, molecules behave as would be expected for an ideal chain [5]. Note that hydrophobicity can increase with temperature. As a result, temperature-induced unfolding is not achievable for many globular proteins, which will denature, and their unique three-dimensional structure shielding hydrophobic core from solvent would be lost. Under such conditions, unprotected hydrophobic patches would be engaged in intermolecular interactions, and as a result, many globular proteins would aggregate and precipitate out of the solution.

Proteins constitute a special type of polymer molecule. Unlike conventional polymers which are built of identical monomeric segments, polymers are built of AAs with different levels of hydrophobicity. Folding of a polymer molecule in water is driven mostly by hydrophobic forces, so that hydrophilic AAs tend to end up at the outside surface of the globule, while hydrophobic AAs are at its centre. Most globular proteins have a unique tertiary three-dimensional structure when folded. The tertiary structure consists of standard secondary structure elements, such as α-helices and β-sheets.

In addition, there are so-called ‘super-secondary’ structures or motifs, which constitute an intermediate level between the secondary and tertiary structures. Thus, the β-α-β motif frequently connects two parallel β-strands while the β-hairpin motif consists of two β-strands joined by a small loop. Several motifs pack together to form domains, self-stabilizing units between 50 and 250 AAs that fold independently from the rest.

The unfolded or denatured protein molecules constitute a one-dimensional sequence of AAs, which is called the primary structure. For unfolded proteins, the scaling exponents tend to converge to values similar to those of polymer molecules in good solvents, such as *α* = 0.62 ± 0.03 at high denaturant concentrations [7]. Scaling exponents reported for folded proteins are often close to *α* = 0.46 ± 0.05 [7] or to *α* = 0.4 [8].

## 3. Diffusion and ergodicity

The scaling of protein length is closely related to two other phenomena: diffusion and solubility. While scaling of the molecular length is performed in the spatial domain, molecular diffusion occurs in the temporal domain. In the classical Einstein–von Smoluchowski model of diffusion, mean displacement is proportional to the square root of the lag time, 〈*r*〉 ∝ *t*^1/2^. Such dependency is inherent for the simple random walk or for the Brownian motion of colloidal particles dissolved in water.

The anomalous diffusion results in the dependency which has the form of a power law

3.1 $$\langle r\rangle \propto t^{\alpha }.$$

The values of the scaling exponent *α* < 1/2 constitute the common case of the subdiffusion [5,6].

The causes for the anomalous diffusion include the macromolecular crowding, flowing through obstacles with a certain density, the ‘hydrodynamic memory’ when a particle's effective mass should be adjusted due to the deceleration caused by incessantly new vortices diffusing slowly through the fluid, as well as turbulent and fractal trajectories of motion. In many of these cases, the damping force depends on the entire history of the particle's trajectory, and it is related to the fractal nature of a turbulent trajectory [9].

The parallelism of the dynamical system's behaviour in the spatial and temporal domains is related to ergodicity. Ergodicity is a property of dynamical systems, which implies the equivalence of the phase space and time averages. Instead of the phase space averages, regular spatial averages can often be used as well as averaging by an ensemble of many particles. This makes ergodicity a crucial property for experimental measuring systems parameters when sufficiently long observations are not practical and are substituted with finite time measurements of many particles [10–12]. The biophysical transport of liquids such as blood, complex biological media flowing in nulcleoplasm, cytoplasm, through cellular membranes, or extracellularly is an area where ergodicity breaking is particularly important [13–15].

The anomalous diffusion leads to the transport deceleration when compared with the classical diffusion law and eventually it results in the ergodicity breaking. It is remarkable that ergodicity breaking can be associated with fractal behaviour, or, in other words with the scaling behaviour characterized by non-integer scaling exponents [10,16]. This general property involves such diverse situations as fractal branching of vascular capillaries [10], random diffusion and the so-called ‘dissipative anomaly’ in the turbulent flow when the dissipation does not approach zero even at the zero viscosity limit so that fractal trajectories lead to deceleration [17,18]. While subdiffusion (0 < *α* < 1/2) is typical for non-motile cells, superdiffusion (1/2 < *α* < 1) is characteristic for motile cells, when intracellular particle motion is superimposed by the locomotion of the cell body [19].

Non-ergodic systems evolve with time, which affects their ability to attain microstates with equal probability, while ergodic systems have no memory of their previous history, and attain all available microstates. Many fundamental ideas in physics of the 20th Century, such as spontaneous symmetry breaking and phase transitions, were related to the concept of ergodicity. For example, it was shown that phase transitions are impossible in finite systems returning to their initial position over a sufficiently long period [20]. Ergodicity also has implications for computational aspects of a dynamical system's behaviour, e.g. identifying Lagrangian Coherent Structures in fluid flow [21]. Non-ergodic behaviour can be compensated by considering the Lamperti transformation [12] or by introducing the ergodicity defect measure.

Moreover, temporal and spatial scaling behaviours have implications for the energetic and informational capacity of living cells [22,23], while topological and dimensional properties are related to their information content [24–26]. During folding, within a very short time (nanoseconds), the chain transforms from a one-dimensional coil to a stable three-dimensional compact globule, passing through the intermediate states. The intermediate states can be stabilized by attenuating the solubility of the polymer molecule, which can be achieved by changing temperature or denaturant concentration. According to the Flory theory, the solubility depends on the entropy of mixing, since the Gibbs free energy change is given by the balance of the enthalpic Δ*H* and entropic Δ*S* contributions ΔG=ΔH−TΔS, and thus by changing temperature the solubility can be controlled. Therefore, there is an equivalence of temporal and spatial behaviour of the molecules with the solubility-controlled behaviour (table 2). In the consequent sections, we will study how scaling and topological properties are related to folding kinetics and molecular interactions.

**Table 2.** The equivalence of temporal, spatial and solubility domains for scaling properties.

phenomenon	time-domain	space-domain	solubility domain
	diffusion	folding	dissolution
*α* < 1/2	subdiffusion (non-ergodic). Due to molecular crowding, hydrodynamic memory, or fractal trajectories	globule, compact three-dimensional structure	below critical, non-soluble, enthalpy dominates over entropy of mixing
*α* = 1/2	random walk, ergodic	ideal chain	theta-point
*α* > 1/2	anomalous superdiffusion (non-ergodic). Lévy flight, motile cells	coil, linear one-dimensional chain	above critical, soluble, entropy of mixing dominates over enthalpy

## 4. Effect of topological properties on folding and molecular interaction

In this section, we will discuss how spatial topological properties, such as the fractional dimension of a molecule, are related to the temporal properties of molecular kinetics during folding and how they affect molecular interactions.

The parameter which is often used to characterize polymer molecules including proteins is the radius of gyration. The radius of gyration of a body about the axis of rotation is defined as the radial distance to a point that would have a moment of inertia the same as the body's actual distribution of mass, if the total mass of the body were concentrated there. It is defined as

4.1 $$R_{g}=\frac{1}{N}\overset{N}{\underset{k=1}{\sum }}{\left(r_{k}-r_{C}\right)}^{2},$$

where $r_{k}$ is the position of the *k*-th segment and $r_{C}$ is the mean position. For a stretched molecule, the radius of gyration is given by

4.2 $$R_{g0}=\frac{1}{\sqrt{12}}L=\frac{1}{\sqrt{12}}aN,$$

and thus *α* = 1. For the ideal chain *α* = 1/2, and the radius of gyration is given by

4.3 $$R_{g}=\frac{1}{\sqrt{6}}a\sqrt{N}.$$

For a spherical globular molecule with *α* = 1/3, the radius of gyration can be estimated as that of a sphere of the radius *R*,

4.4 $$R_{g}=\sqrt{\frac{2}{5}}R=\sqrt{\frac{2}{5}}\sqrt[{3}]{\frac{3Nv}{4}}.$$

Furthermore, for a partially folded chain, one can write

4.5 $$R_{g}=kaN^{\alpha }.$$

The value of the coefficient *k*(*α*) is not defined from geometrical considerations in the general case except for $k(1)=1/\sqrt{12}$ (equation (4.2)) and $k(1/2)=1/\sqrt{6}$ (equation (4.3)); however, an extrapolated dependency could be assumed

4.6 $$k(\alpha )=\frac{1}{\sqrt{12\alpha }}.$$

This immediately yields the dependency

4.7 $$R_{g}=kaN^{\alpha }=R_{g0}\frac{1}{\sqrt{\alpha }}N^{\alpha -1},$$

or

4.8 $$\ln\frac{R_{g}}{R_{g0}}=-0.5\ln\alpha +(\alpha -1)\ln N.$$

When data on the ratio of $R_{g}/R_{g0}$ is available, one can estimate the value of *α* from equation (4.8).

On the basis of the scaling arguments, one can treat a completely unfolded stretched molecule as a one-dimensional object. For such an object, the radius of gyration is proportional to the end-to-end distance and to the number of segments, hence *α* = 1. On the other hand, the completely folded globular molecule can be viewed as a compact three-dimensional object. Its radius of gyration is proportional to the cubic root of the number of segments and to the cubic root of the stretched end-to-end distance, hence *α* = 1/3. During folding, the molecule passes the entire range of scaling exponents, 1/3 < *α* < 1, which corresponds to the transition from the one-dimensional to the three-dimensional state. A fractional molecular dimensionality can be introduced as *D* = 1/*α*.

Most proteins, however, are not completely folded and thus *α* > 0.33. The value of *α* = 1/2 (or *D* = 2) corresponds to the so-called theta-point when the chain behaves like the ideal chain, which can also be interpreted as an effective two-dimensional configuration. Molecular interactions of proteins strongly depend on the radius of gyration and on the dimensionality.

Protein folding is a complex process, and its understanding involves several difficult problems. According to the generally accepted ‘Anfinsen's dogma’, the AA sequence of a protein (i.e. protein's primary structure) completely determines its folded (native) structure [27]. However, it is not clear how protein achieves this native structure and why folding speed is relatively high given a huge number of possible microstates through which the molecule can pass on the way to the unique native state. The fast kinetics of folding constitutes the so-called Levinthal paradox: the number of variants is so large, that thermally activated driven kinetics would take huge amounts of time for a protein to fold [28]. The paradox is usually explained by the hierarchical nature of the folding process, with secondary and super-secondary structures and domains constituting intermediate states between coil and globular proteins.

Naganathan & Muñoz [29,30] showed that the folding time tends to scale with the number of residues (segments) as

4.9 $$\ln T\propto N^{1/2}.$$

Despite that, there are indications that the folding speed strongly depends on the AA sequence even in small structures such as β-sheets [31]. The driving force behind folding is the self-organized critical behaviour caused by hydrophobic interactions [32]. There are numerous indications that folding kinetics is defined by the structural topological properties of proteins [33–36]. Rajasekaran *et al*. [37] studied the distance for which the information on perturbation is transmitted within a molecule. They found universal dependencies in propagation patterns of perturbations into the protein structure as a function of the radius of gyration. In the next section, we will investigate topological properties during scaling of a very short protein whose folding had been investigated thoroughly.

## 5. Case study: small molecule of the Trp-cage protein

**Figure 1.**
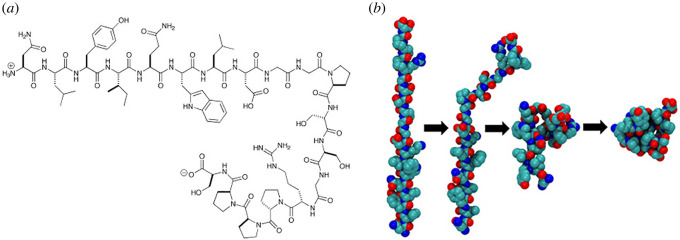
Trp-cage protein molecule (*a*) structural formula and (*b*) schematic of folding.

Trp-cage is a 20-residue miniprotein (sometimes viewed as a polypeptide), which is believed to be the fastest folder known so far (figure 1). The name is due to the Trp burial in the hydrophobic core and due to the cage-like shape of the globular structure. Due to its small size, statistical properties dependent on the number of AAs cannot be studied using this protein. However, despite its small size, Trp-cage shares several features with larger globular proteins. Although the protein has been intensively studied experimentally and with MD simulations, its folding mechanism is not yet fully understood, since some observations suggest a two-state behaviour, while others point to the presence of intermediates [38,39].

MD simulations of the folding/unfolding thermodynamics and kinetics of the Trp-cage showed features similar to globular proteins with increasing hydrostatic pressure destabilizing the native fold [40]. Zhou [41] suggested that the super-fast folding of the TRP-cage is explained by a two-step mechanism with an intermediate metastable state where two correctly formed partial hydrophobic cores are separated by an essential salt bridge between residues Asp-9 and Arg-16 near the centre of the molecule [42].

The AA sequence of the Trp-cage is Asn-Leu-Tyr-Ile-Gln-Trp-Leu-Lys-Asp-Gly-Gly-Pro-Ser-Ser-Gly-Arg-Pro-Pro-Pro-Ser (NLYIQWLKDGGPSSGRPPPS) and its melting point is at *T_m_* = 42°C. However, Barua *et al*. [43] reported that mutations in the helical portion of the protein (replacement of Leu, Ile, Lys or Ser residues by Ala) result in the increase of the melting point to *T_m_* = 64°C. They also found that specific Pro/Trp interactions are not essential for core formation, while the Y3/P19 staple interaction as well as the Trp burial is essential, as the former defines the folding motif as an 18-residue unit. Other stabilizing features that have been identified include a solvent-exposed Arg/Asp salt bridge (3.4–6 kJ mol^−1^) and a buried H-bonded Ser side chain (≈10 kJ mol^−1^) [43].

We initiated our MD simulations using the Trp-Cage AA sequence with an extended initial conformation built by the LEaP module of AMBER [44]. The linear conformation of this protein was designed using the Avogadro software [45]. The three-dimensional molecular structure (PDB ID: 1L2Y) of trip-cage determined by the solution nuclear magnetic resonance (NMR) method as a set (*n* = 38) of stable conformation with the root-mean-square deviation (RMSD) value of 0.32 Å was obtained from the RCSB Protein Data Bank. All MD simulations were fully unrestrained and carried out in the canonical ensemble using the SANDER module available for the Linux/Unix. The MD simulations have included minimization (500 cycles), heating (50 ps) and equilibration (production) phases (50 ns) at 325 K. The Berendsen thermostat was implemented for temperature control and the SHAKE algorithm to constrain the length of covalent bonds, including the hydrogen atoms [46]. The ff99 force field was used as it was previously employed for similar modelling [47]. Solvation effects were incorporated using the Generalized Born model, as implemented in AMBER [48]. The Rosetta crystallographic refinement protocol was implemented to assess the conformational stability of the NMR structure [49]. The ColabFold and PEP-FOLD3 protocols were applied to perform *ab initio* protein folding of Trp-Cage using either the AlphaFold2 and RoseTTAFold algorithms or the structural alphabet together with a greedy algorithm and a coarse-grained force field [50–52].

**Figure 2.**
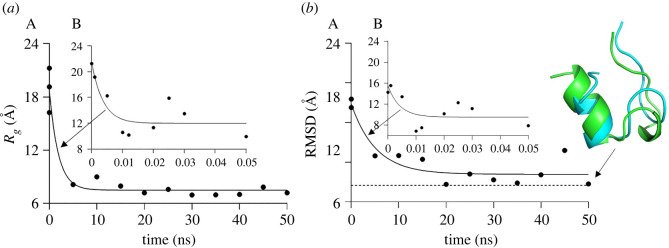
Two stages of Trp-cage folding, the equilibration (A) and heating (B) phases, showing (*a*) the radius of gyration and (*b*) RMSD of the simulated (blue) from the reference configuration (green) versus time.

The MD simulation of folding data is shown in figure 2*a*. The folding process involves two stages. The first stage is the heating phase, which lasts for less than 50 ps, and is followed by the equilibration stage lasting for about 50 ns. During the heating phase, the molecule undergoes significant structural changes due to heating disturbances. The radius of gyration of the protein decreases from *R_g_* = 21.25 Å to *R_g_* = 10.6 Å in 10 ps, with some oscillations between 10 Å and 16 Å lasting for about 50 ps during the entire heating stage. After that, it takes a much longer time to decrease to the minimum value of *R_g_* ≈ 6.97 Å at 5 ns, and the molecule oscillates at about that value during the equilibration stage.

The RMSD of the molecule from its final exact folded reference shape (measured experimentally by the structural analysis) also undergoes significant fluctuations during folding (figure 2*b*). At the heating stage, the RMSD oscillates between 16 Å and about 7 Å. During the equilibration, it further decreases but stays above the level of 3 Å during the entire period of 50 ns. This shows that the folding does not complete at 50 ns. Given that the folding is initiated at the C-terminus of the molecule, which is very compliant and flexible, the complete folding would need much larger tome of about 4 µs, as the RMSD value of 0.56 Å (close to the ideal 0.5 Å) as found with the AlphaFold2 algorithm [53].

**Figure 3.**
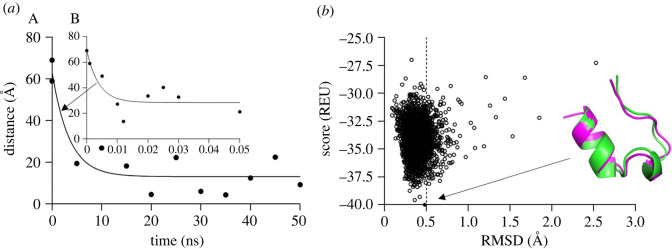
(*a*) End-to-end distance versus time during the heating (A) and equilibration (B) phases of folding. (*b*) Energy versus RMSD of the simulated (red) from the reference configuration (green) for the TRP-cage molecule. The best-fit configuration is shown by an arrow.

The end-to-end distance decreases from *L* = 73.61 Å down to about 9 Å as shown in figure 3*a*. Similar to *R_g_*, it oscillates significantly during both the heating and equilibration stages. The plot figure 3*b* shows the protein deviation from its reference configuration using the Rosetta refinement protocol with Rosetta energy units to find the conformation with minimal energy (the black spot), which shows the RMSD of 0.5 Å. The refinement protocol shows the deviation at about ±2.5 Å. The MD simulation protein folding is close to this threshold (3 Å in figure 2*b*); however, the data demonstrate that the MD configuration at 50 ns is not yet the ideal folding [54].

**Figure 4.**
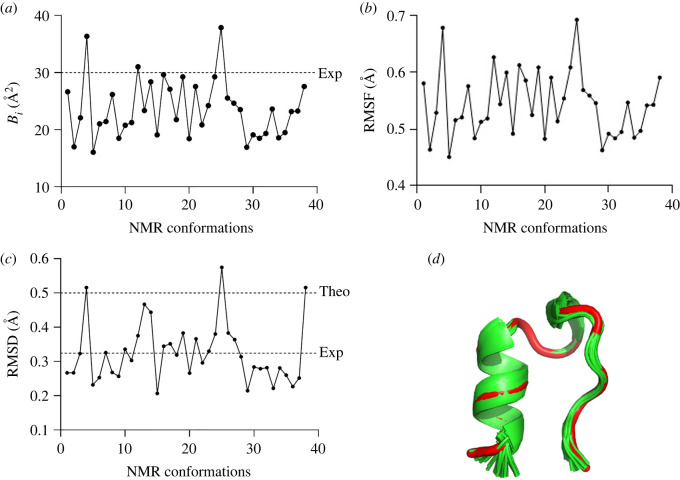
Calculated values of (*a*) temperature value (*B_i_*), (*b*) RMSF, (*c*) RMSD versus NMR conformations and (*d*) three-dimensional alignment of the NMR conformations. The reference structure as the first conformation is shown as a tube model and coloured in red. The experimental (Exp) and theoretical (Theo) thresholds are depicted as dashed lines.

The experimental parameters as a function of the NMR conformations are shown in figure 4.

Additionally, the structural analysis of NMR structures of TRP-cage in the solution has shown the conformational stability of this protein judging by the temperature value (*B*_i_). This parameter was determined to be less than 30 Å for most conformations, which signifies high confidence in the TRP-cage atomic positions. On the other hand, the *B*_i_ value of greater than 60 signifies disorder (figure 4*a*). These data are also in agreement with the RMSF and RMSD values, indicating a relatively small deviation of the NMR models from the reference structure (first conformation) (figure 4*b–d*). Moreover, most of the NRM conformations possessed much lower RMSD on average (RMSD = 0.33 Å) than for the model predicted previously by the Rosetta refinement protocol (RMSD ≈ 0.5 Å).

Using the values of *N* = 20, *L* = 73.61 Å and *a* = *L*/*N* = 3.68 Å, the power exponents and dimensionality of the molecule can be estimated from equation (4.8). For the non-intersecting chain (*α* = 0.6), this yields *R_g_* = 8.28 Å, while for the ideal chain (*α* = 1/2) *R_g_* = 6.73 Å, and for the globule (*α* = 0.33) *R_g_* = 4.99 Å. Note that a rough estimate of the total molecular volume is given by *V* = *a*^3^*N* = 997 Å^3^, which corresponds to the sphere with a radius of 6.19 Å or to *R_g_* = 4.8 Å. Since the shape of the actually folded molecule is not perfectly spherical, the radius of gyration of the simulated folded molecule, *R_g_* ≈ 6.97 ± 3.5 Å, is larger than the estimated values of *R_g_* = 4.99 Å and *R_g_* = 4.8 Å. The value of *R_g_* ≈ 6.97 corresponds to *α* = 0.52. For the dimensional analysis, *D* = 1/*α*, one can conclude that during folding the molecule passes the stages between 1 ≤ *D* ≤ 1.92.

**Figure 5.**
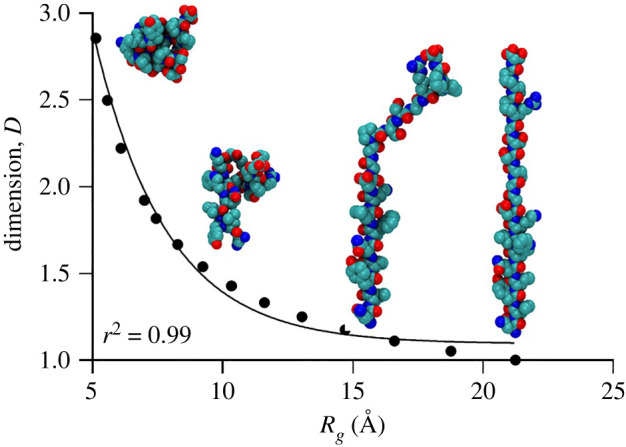
Dimensionality versus the radius of gyration during folding.

Figure 5 presents the dimensionality of the chain as a function of the radius of gyration from equation (4.8). During folding, the chain transforms from the one-dimensional linear or coil structure to a three-dimensional globule. It is observed that the dimension changes from *D* = 1 to approximately *D* ≈ 2 during the simulated stages of folding. The pure globular stage (*D* = 3) is not achieved. As it was discussed, the MD configuration at 50 ns is not yet the ideal folding. Moreover, even the ideal reference configuration of the TRP-cage molecule (e.g. green in figure 3*b*) is not an ideal globule, having a ‘tail’ and a ‘cage’ (which gave the name to this molecule). Despite that, it is seen that dimensional topological parameters can be calculated and they can provide new insights or at least a new point of view on the molecular configuration and its interactions.

## 6. Conclusion

Topological characteristics of molecular chains include their scaling exponents which may be interpreted as inverse dimensionality *D* = 1/*α*. The scaling exponents at different stages of folding of the TRP-cage molecule were estimated from the radius of gyration of the molecules. During folding, a molecule goes through a continuous transition from a one-dimensional linear chain to a three-dimensional compact globule. Intermediate states are interpreted as having the fractional dimensionality between one- and three-dimensional. While folding is a dynamic process developing in the time domain, the intermediate states with a certain specific fractional dimension are structures in the space domain. However, structures with fractional dimensions are geometrically equivalent to the trajectories of particles driven by diffusion, which are patterns in the temporal domain. Moreover, the equilibrium states can be tuned by changing solubility in such a way that the equilibrium state becomes one of the fractional dimensionality states.

Topological properties are correlated with molecular interaction energy. Thus, by tuning molecular solubility, one can, in principle, modify the ligand–receptor interaction properties of large biomolecules. While tested only on a small model Trp-cage protein, it is expected that the same considerations apply to larger molecules. However, many natural functional proteins could be folded to a different degree, and many others could contain IDRs, which also could be folded to a different degree, suggesting that considered topological (dimensional) scaling should be adjusted. The considered model represents a simplification of protein folding. Natural proteins in aqueous environments are never linear stretched molecules, and even in the highly unfolded states (e.g. in the presence of strong denaturants), they are never random coils due to their heterpolymeric nature and always contain some residual structure. A computational method of topological bioscaling analysis can be developed using these ideas.

## Data Availability

Data available from the Dryad Digital Repository: https://doi.org/10.5061/dryad.8gtht76rn [54].
